# Publisher Correction: Decision support for the quickest detection of critical COVID-19 phases

**DOI:** 10.1038/s41598-021-91775-2

**Published:** 2021-07-28

**Authors:** Paolo Braca, Domenico Gaglione, Stefano Marano, Leonardo M. Millefiori, Peter Willett, Krishna Pattipati

**Affiliations:** 1 Research Department, NATO STO Centre for Maritime Research and Experimentation, 19126 La Spezia, Italy; 2 Department of Information & Electrical Engineering and Applied Mathematics (DIEM), University of Salerno, 84084 Fisciano, SA Italy; 3 Department of Electrical and Computer Engineering, University of Connecticut, Storrs, 06269 USA

Correction to: *Scientific Reports* 10.1038/s41598-021-86827-6, published online 20 April 2021

The original version of this Article contained an error in the copyright line.

“© The Author(s) 2021”

now reads:

“© NATO 2021”

The original Article has been corrected.

