# Supplementary material for: Decision support for the quickest detection of critical COVID-19 phases
Source: Sci Rep. 2021 Apr 20;11:8558. doi: 10.1038/s41598-021-86827-6 (PMC8058081; doi:10.1038/s41598-021-86827-6)
Supplement: Supplementary file 1 — Supplementary Information 1. [file 41598_2021_86827_MOESM1_ESM.pdf]

Decision Support for the Quickest Detection of Critical COVID-19 Phases
— Supplementary Information —

Paolo Braca[1,*], Domenico Gaglione[1], Stefano Marano[2], Leonardo M. Millefiori[1], Peter Willett[3], and Krishna Pattipati[3]

[1]NATO STO Centre for Maritime Research and Experimentation, Research Department, La Spezia, 19126, Italy
[2]University of Salerno, Department of Information & Electrical Engineering and Applied Mathematics (DIEM), Fisciano (SA), 84084, Italy
[3]University of Connecticut, Department of Electrical and Computer Engineering, Storrs, 06269, USA
[*]Corresponding author: paolo.braca@cmre.nato.int

ABSTRACT

In this document, we provide some mathematical details and analysis related to the application of the Mean-Agnostic Sequential Test (MAST) quickest-detection procedure to COVID-19 infection data.

Observation Model

Classical Deterministic SIR

Let us start, for the sake of simplicity, from the classical Susceptible-Infectious-Recovered (SIR) model of epidemic spread; a similar discussion holds for more complicated compartmental models. There are P individuals (for concreteness, this may be the population of a state or a country), grouped into three classes, or *compartments*: susceptible $S(t)$, infected $I(t)$, and removed (or recovered) $R(t)$. Let $s(t) = S(t)/P$, $i(t) = I(t)/P$ and $r(t) = R(t)/P$, denote the corresponding fractions of individuals. Each susceptible individual infects, on the average, β randomly-chosen other individuals per unit of time. Each infected individual will then recover (or, unfortunately, pass away) at a constant average rate γ. The popular SIR model[1–3] is formalized mathematically as follows

$$\begin{cases} \frac{ds(t)}{dt} &= -\beta\, s(t) i(t), \\ \frac{di(t)}{dt} &= \beta\, s(t) i(t) - \gamma i(t), \\ \frac{dr(t)}{dt} &= \gamma i(t), \end{cases} \tag{1}$$

with initial conditions $r(0) = 0$, $s(0) = 1 - i(0)$; $i(0)$ is a (fairly small) fraction of the total population that gives rise to the spread of the infection. Note that one of the three equations in (1) is redundant, in view of the conservation constraint $r(t) + s(t) + i(t) = 1$. Dividing the first of (1) by $s(t)$ and solving for $i(t)$ from the third equation, one gets $d \log s(t) = -\frac{\beta}{\gamma}\, dr(t)$. The quantity β/γ goes also under the name of *contact number* and is a combined characteristic of the population and the disease. Also, using $i(t) = 1 - r(t) - s(t)$ in the third equation, we eliminate $i(t)$ and arrive at

$$\begin{cases} s(t) &= s(0) e^{-\frac{\beta}{\gamma} r(t)}, \\ \frac{dr(t)}{dt} &= \gamma \Big[1 - r(t) - s(0) e^{-\frac{\beta}{\gamma} r(t)} \Big], \end{cases} \tag{2}$$

to be solved numerically. At the onset of the epidemic, i.e., for $t \to 0$, the fractions of susceptible and recovered are approximately constant and equal to $s(t) \approx 1$ and $r(t) \approx 0$, respectively. As $t \to \infty$, if a steady-state regime for $r(t)$ emerges, we expect $\frac{dr(t)}{dt} = 0$. Imposing this condition and denoting by $r(\infty) = \lim_{t\to\infty} r(t)$ and $s(\infty) = \lim_{t\to\infty} s(t)$, from the second of (2), we have $r(\infty) = 1 - s(0) e^{-\frac{\beta}{\gamma} r(\infty)} \approx 1 - e^{-\frac{\beta}{\gamma} r(\infty)}$, which can be numerically solved for $r(\infty)$. An analytical solution is also

available, in terms of the so-called Lambert W function (also known as product logarithm):[4]

$$r(\infty) = \frac{W\left(-\frac{\beta}{\gamma}e^{-\frac{\beta}{\gamma}}\right)+\frac{\beta}{\gamma}}{\frac{\beta}{\gamma}}. \tag{3}$$

From the first of (2), we see that the fractions of recovered and susceptible individuals reach steady-state constant values $r(\infty)$ (typically > 0) and $s(\infty)$ (< 1) verifying $r(\infty) = 1 - s(\infty)$, while $i(\infty) = \lim_{t\to\infty} i(t) = 0$. Suppose $s(t) \approx s(t^\star)$, for some $t^\star$. For instance, this happens at the beginning of the epidemic with $s(t^\star) = s(0) \approx 1$, and at the end of the epidemic, with $s(t^\star) = s(\infty) < 1$. Thus, at different stages of the epidemic, $s(t)$ can be approximately assumed constant over short intervals of time. We make the assumption that variations of $s(t)$ can indeed be neglected over short intervals of time. With $s(t) = s(t^\star)$, from the second equation in (1), we get

$$\frac{di(t)}{dt} = \alpha\, i(t) \quad \Rightarrow \quad i(t) = i(0)e^{-\alpha t}, \tag{4}$$

with $\alpha{:=}\beta s(t^\star) - \gamma$. Usually, $\beta - \gamma > 0$, while $\beta s(\infty) - \gamma < 0$, so that $i(t)$ initially grows exponentially at rate $\beta - \gamma$ and eventually decreases exponentially to zero at rate $\beta s(\infty) - \gamma$.

Proposed Model

Epidemic data are typically collected on a daily basis, and a discrete version of (4) with discretization step $\Delta t = 1$ day can be obtained as follows. Let n be the day index. With the obvious notation $i_n = i(n\Delta t)$ and similarly for other quantities, the differential equation on the left-hand side of (4) is approximated by $\frac{i_{n+1}-i_n}{\Delta t} = i_{n+1} - i_n = \alpha\, i_n$. This yields

$$\Delta i_n{:=}i_{n+1} - i_n = \alpha\, i_n \quad \Rightarrow \quad i_n = i_0(1+\alpha)^n, \quad n \geq 1, \tag{5}$$

for some $i_0 > 0$, and therefore the ratio (also known as growth, for $\alpha > 0$, or decay, for $\alpha < 0$, rate)

$$x_n{:=}\frac{i_n}{i_{n-1}}, \quad n \geq 1, \tag{6}$$

is constant and equal to $(1+\alpha)$. It is useful to bear in mind that we typically have $|\alpha| \ll 1$. From (5) we see that the sequence i_n grows or decays exponentially according to the sign of α, and its evolution can be locally assumed to be linear in n because $(1+\alpha)^n \approx 1 + n\alpha$, in view of $|\alpha| \ll 1$. For later use, note that, from the third equation in (1), we also get

$$\Delta r_n{:=}r_{n+1} - r_n = \gamma i_n. \tag{7}$$

It is evident that real-world data lead to "noisy" versions of the previous expressions. There exist two main sources of uncertainty. The first is implicit in the nature of the contagion, which obviously depends on a number of medical and social factors that are problematic to model in deterministic terms. Accordingly, the sequence $\{x_n\}_{n\geq 1}$ in (6) is better represented by a collection of random variables, leading to the following model for the fraction of infected individuals on day n:

$$i_n = i_{n-1}\, x_n = i_0 \prod_{k=1}^{n} x_k, \qquad n \geq 1. \tag{8}$$

In (8), we assume that $\{x_n\}_{n\geq 1}$ is a sequence of nonnegative *independent* random variables with mean close to unity due to the fact that $|\alpha| \ll 1$. The independence assumption is crucial to ensure mathematical tractability; however, as we show in the section on "Performance Assessment - Synthetic Data," slight deviations from the condition of perfect independence do not significantly affect the performance of the proposed Mean-Agnostic Sequential Test (MAST).

The second source of uncertainty is related to the way in which the epidemic data are collected, as we describe next. First, let us consider the values of "new positives" recorded on day n and its relationship to the "total cases" on days n and $n-1$:

$$p_n{:=}\text{ new positives}_n = \text{total cases}_n \text{ - total cases}_{n-1}. \tag{9}$$

Since the number of total cases on day n is equal to $i_n + r_n$, we have $p_n = i_n + r_n - (i_{n-1} + r_{n-1}) = \Delta i_{n-1} + \Delta r_{n-1} = (\alpha+\gamma)i_{n-1}$, because of (5) and (7). Then, we get

$$\frac{p_{n+1}}{p_n} = \frac{i_n}{i_{n-1}} = x_n, \tag{10}$$

Country	p-Value	σ
Albania	.070	.020
Austria	.424	.025
Belgium	.104	.027
Canada	.030	.018
France	.013	.065
Germany	.017	.023
Hungary	.063	.032
Italy	.105	.015
Netherlands	.117	.016
Norway	.056	.033
Portugal	.623	.017
Spain	.001	.047
UK	.006	.016
US	.662	.006

Table S1. p-value of Kolmogorov-Smirnov goodness-of-fit test, and estimated value of σ.

yielding

$$p_{n+1} = p_n x_n = p_1 \prod_{k=1}^{n} x_k, \qquad n \geq 1, \tag{11}$$

for some initial value p_1. This is important, as while there is a natural modeling in terms of the proportion of infected individuals i_n, the time-sequence $\{i_n\}$ is difficult to obtain as the number that get removed from it (by recovery or death) is often not reported. On the other hand, the number of newly infected p_n is easily available, and reported with vigor. Based on (11), the sequence $\{x_n\}$ is obtained from $\{p_n\}$ using the relationship $x_n = p_{n+1}/p_n$. Returning to the problem of the second source of uncertainty, the sequence of daily new positives that is available from the John Hopkins University[5] is expected to differ from the *actual* numbers of new positives $\{p_n\}_{n\geq 1}$ due to gross errors, missing values or delays in the reported data. For instance, it may happen that, on a given day, the fraction of new positives is not reported (some peripheral data collection unit did not communicate with the central unit in a timely manner) and such unreported fraction is then added to the value observed on a later day. Also, it typically happens that the number of reported cases over the weekend is systematically smaller than the values recorded on other days of the week, due to the smaller number of swabs tested during weekends. Similar periodic modulation effects are already reported in the literature.[3] For these reasons, we first eliminate from the data anomalous values (negative numbers) and then, in place of (11), we consider a more sensible observation model:

$$\widetilde{p}_n = p_n + w_n, \qquad n \geq 1, \tag{12}$$

where w_n is a noise term taking into account the effect of gross errors. Due to their nature, we expect that gross errors are washed away by averaging the data over a time window of length L, where L is of the order of a week or longer. On the other hand, we have seen that the expected behavior of p_n over a sufficiently small time window is approximately linear. For this reason, the downloaded sequence of new positives is first filtered by a moving average filter of length L days (in the paper we use $L = 21$, see below). Under these assumptions, we conclude from (12) that:

$$\langle \widetilde{p}_n \rangle_L = \langle p_n \rangle_L + \langle w_n \rangle_L \approx p_n, \tag{13}$$

where $\langle p_n \rangle_L = \frac{1}{L} \sum_k p_k$ denotes the L-th order uniformly-weighted moving average [MA(L), for short] of the sequence $\{p_n\}$, where the sum involves L entries centered on the n-th sample. Admittedly, the approximation in (13) is rather sharp, but works for our purposes. Summarizing, the sequence of daily new positives (after removing negative entries) is first processed by an arithmetic moving average filter of order L, yielding $\{p_n\}_{n\geq 1}$ and then, from this, the sequence $\{x_n = p_{n+1}/p_n\}$ is obtained as in (10).

Statistical Characterization

Recall that the subscript n to x_n and other quantities denotes the index of the day. The statistical distribution of the sequence $\{x_n\}$ is derived from COVID-19 data made available by the Johns Hopkins University,[5] as described next. For concreteness,

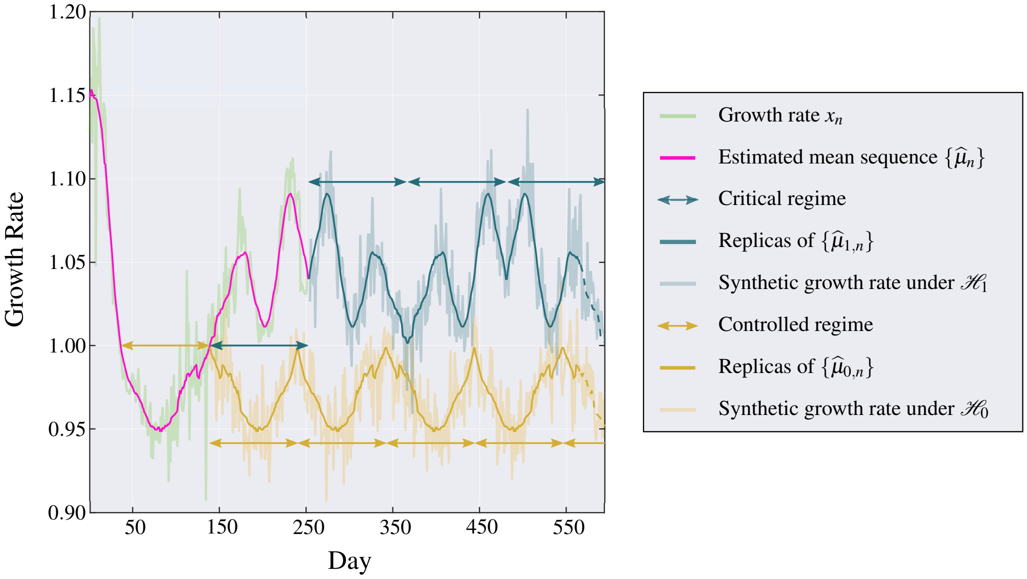

Figure S1. Example of the construction of the periodic counterparts of $\{\widehat{\mu}_{0,n}\}$ and $\{\widehat{\mu}_{1,n}\}$ from the Italian data, used to generate the synthetic grow rates under the controlled and the critical regime, respectively. From the growth rate sequence x_n (green curve), the estimated mean sequence $\{\widehat{\mu}_n\}$ (magenta) is obtained through a uniformly-weighted moving average filter of length 21 days. From $\{\widehat{\mu}_n\}$, the two sub-sequences $\{\widehat{\mu}_{0,n}\}$ (dark yellow) and $\{\widehat{\mu}_{1,n}\}$ (dark cyan) are extracted, so as to verify $\widehat{\mu}_{0,n} \leqslant 1$ and $\widehat{\mu}_{1,n} > 1$. The two sub-sequences are then replicated, with the odd replicas flipped to preserve continuity, yielding the dark yellow and dark cyan solid curves, which are finally used as mean values to generate the synthetic data under the controlled and the critical regime, respectively. One realization of the resulting synthetic growth rates under $\mathscr{H}_0$ and under $\mathscr{H}_1$ is superimposed to the mean values, for illustration purpose.

let us refer to the data from Italy. Figure 2(a) of the main document shows the measured sequence (not normalized to total population) of new positives $\{\widetilde{p}_n\}$ and in green the smoothed version $\{p_n\}$ obtained by MA(21), i.e., as the output of a moving average filter of length $L = 21$ days, fed by $\{\widetilde{p}_n\}$. The corresponding sequence of ratios $\{x_n = p_{n+1}/p_n\}$ is shown in Fig. 2(b) in green. Numerical investigations, not detailed for the sake of brevity, show that the growth rate x_n can be modeled by a Gamma random variable with shape parameter κ and scale parameter θ, with $\kappa\theta$ close to unity. Since κ is on the order of several hundreds, the Gamma distribution is practically indistinguishable from a Gaussian distribution with mean $\mu = \kappa\theta$ on the order of unity, and standard deviation $\sigma = \sqrt{\kappa}\theta \ll 1$. In light of these considerations, we adopt the Gaussian model because it leads to a detector with simple and intuitive structure.

Accordingly, let us reconsider the sequence $\{x_n\}$ shown in green in Figure 2(b). First, we disregard the initial portion of the sequence $\{x_n\}$ that corresponds to the first peak of $\{p_n\}$ (first wave of the epidemic). The omitted data corresponds to values of the day index n smaller than the index of the first passage (from the above) by one of the sequence $\{x_n\}$. Then, we consider the smoothed version $\{\widehat{\mu}_n\}$ of $\{x_n\}$, shown in magenta in Figure 2(b). The smoothing is obtained by processing $\{x_n\}$ again by a MA(21) filter. The length $L = 21$ of the two MA filters used in this analysis have been selected by trial and error. Note that the filters are not *causal*:[6] to compute the output at time n, $(L-1)/2$ successive samples of the input are needed. Note also that the filters are truncated at the endpoints where less than L samples are available.

By subtracting the smoothed version $\{\widehat{\mu}_n\}$ from the sequence $\{x_n\}$, a sequence $\{x_n - \widehat{\mu}_n\}$ with approximately zero-mean is obtained. Then, we verify that the random variables $x_n - \widehat{\mu}_n$, $n = 1, 2, \ldots$, have one and the same standard deviation $\sigma \ll 1$, with good accuracy. In particular, there is no evidence of a substantial change in standard deviation between the regions with $\widehat{\mu}_n \leq 1$ and those in which $\widehat{\mu}_n > 1$. The estimated values of σ for the 14 nations considered in the main document is reported in Table S1. To distinguish between these two regions, we introduce the notations $\mu_{0,n} := \mu_n \leq 1$ and $\mu_{1,n} := \mu_n > 1$ for the actual (unknown) mean values. Note that, since $\mu_{0,n}$ and $\mu_{1,n}$ are close to unity and $\sigma \ll 1$, we see that $x_k < 0$ with negligible probability.

The quantity $\{x_n - \widehat{\mu}_n\}$ so obtained is modeled as a sequence of zero-mean independent Gaussian random variables with (nation-dependent but small) standard deviation σ. The independence assumption is necessary to ensure mathematical tractability and, in practice, slight deviations from the condition of perfect independence do not significantly affect our results. In this respect, it should be also noted that the smoothing operation shown in Eq. (13) enforces correlation over the sequence $\{p_n\}$, and, in turn, over the sequence of ratios $\{x_n = p_{n+1}/p_n\}$.

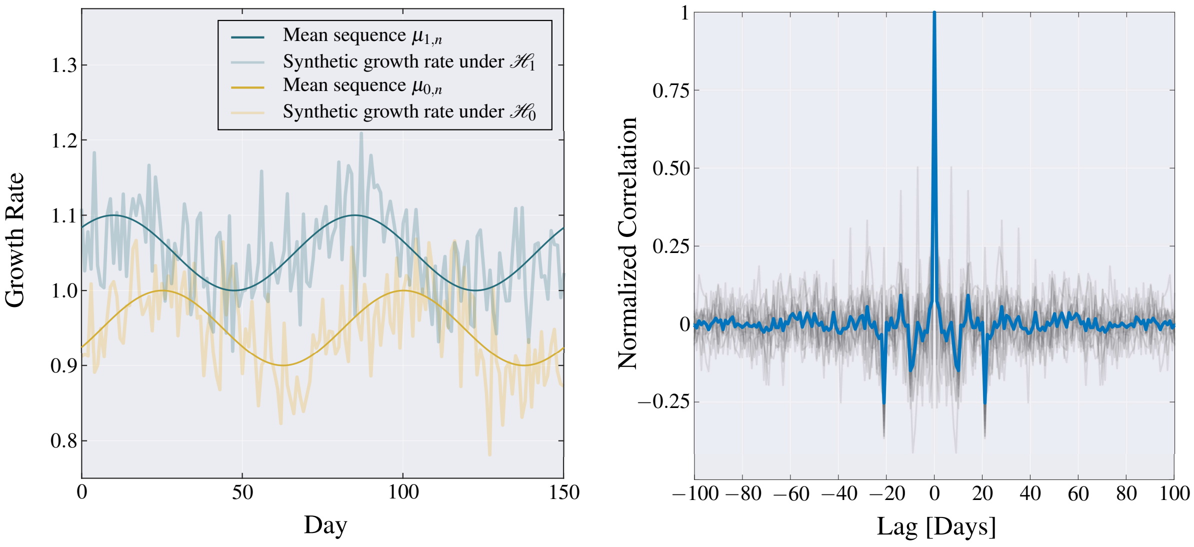

(a) (b)

Figure S2. (a) Mean sequences $\{\mu_{0,n}\}$ (dark yellow) and $\{\mu_{1,n}\}$ (dark cyan) used in the synthetic scenario. These are sinusoidal waves with period of 75 days oscillating between $1-\varepsilon$ and 1 under $\mathscr{H}_0$, and between 1 and $1+\varepsilon$ under $\mathscr{H}_1$, with $\varepsilon = 0.1$. One realization of the resulting synthetic growth rates under $\mathscr{H}_0$ and under $\mathscr{H}_1$ is superimposed to the mean values, for illustration purpose. (b) Normalized correlation (blue line) enforced on the synthetic sequence $\{x_n - \mu_n\}$ used to numerically evaluate the performance of MAST. This normalized correlation is derived by averaging the normalized correlations obtained from the data of the 14 nations considered in the main document; each of these is reported on the background (light grey lines).

Finally, in Table S1, we verify that the p-value obtained by the Kolmogorov-Smirnov (KS) goodness-of-fit test[7] is larger than 0.01, meaning that the Gaussian assumption cannot be rejected at 1% significance level, for almost all the countries (except Spain and UK). We thus arrive at the following model of the observable growth rate sequence $\{x_n\}$: $x_n \sim \mathscr{N}(\mu_{0,n}, \sigma)$ with $\mu_{0,n} \leq 1$, under the controlled regime, and $x_n \sim \mathscr{N}(\mu_{1,n}, \sigma)$ with $\mu_{1,n} > 1$, under the critical one.

Performance Assessment

Real Data

The implementation of the MAST algorithm does not require knowledge of the mean sequences $\{\mu_{0,n}\}$ and $\{\mu_{1,n}\}$ and, in fact, it has been designed just to cope with this uncertainty. However, the performance of the test depends, of course, on the specific scenario under consideration. Performance of MAST run on publicly available COVID-19 infection data from different countries is reported in the main document and obtained as follows. For each country, we investigate the performance of MAST by using the estimates $\{\widehat{\mu}_{0,n}\}$ and $\{\widehat{\mu}_{1,n}\}$ of the mean sequences $\{\mu_{0,n}\}$ and $\{\mu_{1,n}\}$. Since we need to simulate arbitrarily long data streams under both the controlled and critical regimes, we construct periodic counterparts of the sequences $\{\widehat{\mu}_{0,n}\}$ and $\{\widehat{\mu}_{1,n}\}$, as illustrated in Fig. S1. Using these periodic counterparts, we obtain the performance of the test by standard Monte Carlo computer experiments,[2,8,9] involving 10^5 independent runs for each value of the threshold χ. This gives the relationship between R and χ, and between Δ and χ, for relatively small values of χ. The former relationship is almost exactly exponential, and the latter almost exactly linear. Thus, the aforementioned relationships are extrapolated to arbitrarily large values of χ. This explains why the figures of the main document report values of R that would be difficult to obtain by standard computer experiments.

Synthetic Data

The performance of MAST is also evaluated in a synthetic scenario and compared with those of a *naive* Page's test. To emulate a realistic scenario, we assume that the mean sequence $\{\mu_n\}$ has a periodic evolution not exceeding 1 in the controlled regime, and not falling below 1 in the critical regime. Specifically, we set

$$\mu_{0,n} = 1 + \frac{\varepsilon}{2}\left(\cos(2\pi n M^{-1} + \phi_0) - 1\right), \tag{14}$$

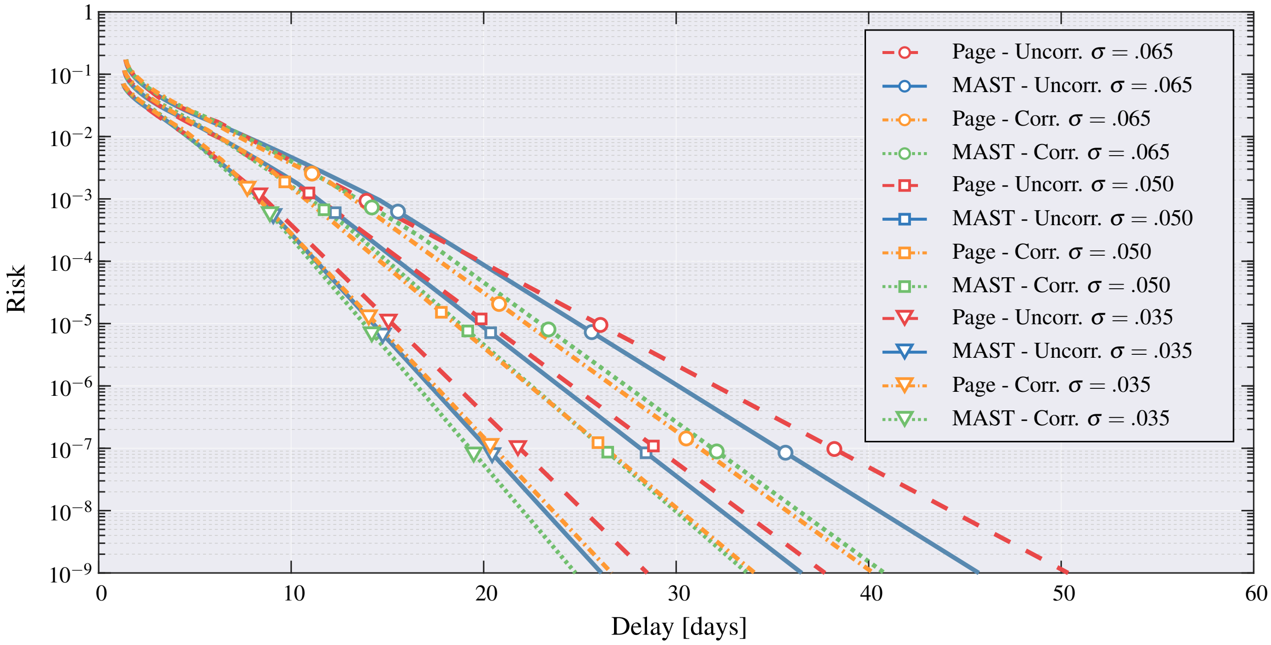

Figure S3. Performance of MAST and the naive Page's test in terms of risk versus mean delay. Red dashed lines and blue solid lines refer to the naive Page's test and MAST, respectively, when run on uncorrelated sequences; yellow dash-dotted lines and green dotted lines refer to the naive Paige's test and MAST, respectively, when applied on correlated sequences. The markers allow to distinguish between different values of σ: circles for $\sigma = 0.065$, squares for $\sigma = 0.050$, and triangles for $\sigma = 0.035$.

and

$$\mu_{1,n} = 1 + \frac{\varepsilon}{2}\left(\cos(2\pi n M^{-1} + \phi_1) + 1\right). \tag{15}$$

Fig. S2(a) shows the mean sequences used for this analysis, with $\varepsilon = 0.1$, period $M = 75$ days, and phases ϕ_0 and ϕ_1 uniformly drawn from $[0, 2\pi)$; in addition, one realization of the resulting synthetic growth rates under $\mathscr{H}_0$ and under $\mathscr{H}_1$ is provided, obtained by adding to each mean sequence a zero-mean Gaussian noise process with standard deviation $\sigma = 0.050$. Moreover, in order to numerically demonstrate that slight deviations from the condition of perfect independence of the sequence $\{x_n - \mu_n\}$ do not significantly affect the performance of the MAST (as claimed above), we also consider the case of a correlated zero-mean Gaussian noise process. The (normalized) correlation we enforce on the sequence $\{x_n - \mu_n\}$ is shown in blue in Fig. S2(b); this is obtained by averaging the correlations computed from the sequences $\{x_n - \widehat{\mu}_n\}$ of the 14 nations considered in the main document. The correlated Gaussian process is generated by filtering an uncorrelated Gaussian sequence as described in [10, Ch. 11.3.2].

MAST is compared with a naive Page's test that is aware of the bounds of the mean sequence under each hypothesis, namely, the lower bound $1 - \varepsilon$ and the upper bound 1 under $\mathscr{H}_0$, and the lower bound 1 and the upper bound $1 + \varepsilon$ under $\mathscr{H}_1$; naive here implies that the test does not know the exact evolution of the mean sequence, but only its extreme values. The naive Page's test is based on Eq. (6), reported in the main document, imposing $\alpha = \varepsilon$, and corresponds to the standard Page's test when $M = 1$, $\phi_0 = \pi$, $\phi_1 = 0$, and the data are uncorrelated.

Fig. S3 presents the performance of MAST and the naive Page's test in terms of risk versus mean delay for different values of σ, obtained by standard Monte Carlo computer experiments[2,8,9] involving 10^5 independent runs for each value of the threshold χ. We observe that in the case of uncorrelated sequences, MAST outperforms the naive Page's test, even though the former does not have any prior information about the mean sequences, except for them being above or below 1, that is, $\mu_{0,n} \leqslant 1$ and $\mu_{1,n} > 1$. By enforcing the correlation, the performance of both MAST and the naive Page's test improves; however, there are no remarkable differences, with the displacement in terms of mean delay being no more than 1 day for the MAST and 2 days for the naive Page's test at a risk level of 10^{-4}.

References

1. Kermack, W. O., McKendrick, A. G. & Walker, G. T. A contribution to the mathematical theory of epidemics. *Proc. R. Soc. Lond* **115**, 700–721, DOI: 10.1098/rspa.1927.0118 (1927).
2. Allen, L. J. A primer on stochastic epidemic models: Formulation, numerical simulation, and analysis. *Infect. Dis. Model.* **2**, 128–142, DOI: 10.1016/j.idm.2017.03.001 (2017).
3. Gaglione, D. *et al.* Adaptive Bayesian learning and forecasting of epidemic evolution - Data analysis of the COVID-19 outbreak. *IEEE Access* **8**, 175244–175264, DOI: 10.1109/ACCESS.2020.3019922 (2020).
4. Wang, F. Application of the Lambert W function to the SIR epidemic model. *The Coll. Math. J.* **41**, 156–159, DOI: 10.4169/074683410x480276 (2010).
5. Johns Hopkins University Center for Systems Science and Engineering (JHU CSSE). COVID-19 data repository. https://github.com/CSSEGISandData/COVID-19 (2020).
6. Oppenheim, A. V. & Willsky, A. S. *Signals & Systems* (Prentice Hall International, Inc., Apple Saddle River, New Jersey, 1997), 2 edn.
7. Feller, W. On the Kolmogorov-Smirnov limit theorems for empirical distributions. *Ann. Math. Stat.* **19**, 177–189, DOI: 10.1214/aoms/1177730243 (1948).
8. Kay, S. M. *Fundamentals of Statistical Signal Processing, Volume II: Detection Theory* (Prentice Hall PTR, Upper Saddle River, NJ, USA, 1998).
9. Lehmann, E. L. *Testing Statistical Hypotheses* (Springer, 2005).
10. Oppenheim, A. & Verghese, G. *Signals, Systems and Inference* (Pearson, Hoboken, NJ, USA, 2016).
