# Supplementary figures and images for: Decision support for the quickest detection of critical COVID-19 phases
Source: Sci Rep. 2021 Apr 20;11:8558. doi: 10.1038/s41598-021-86827-6 (PMC8058081; doi:10.1038/s41598-021-86827-6)

MAST Dashboard: <https://covid-mast.github.io/#/>

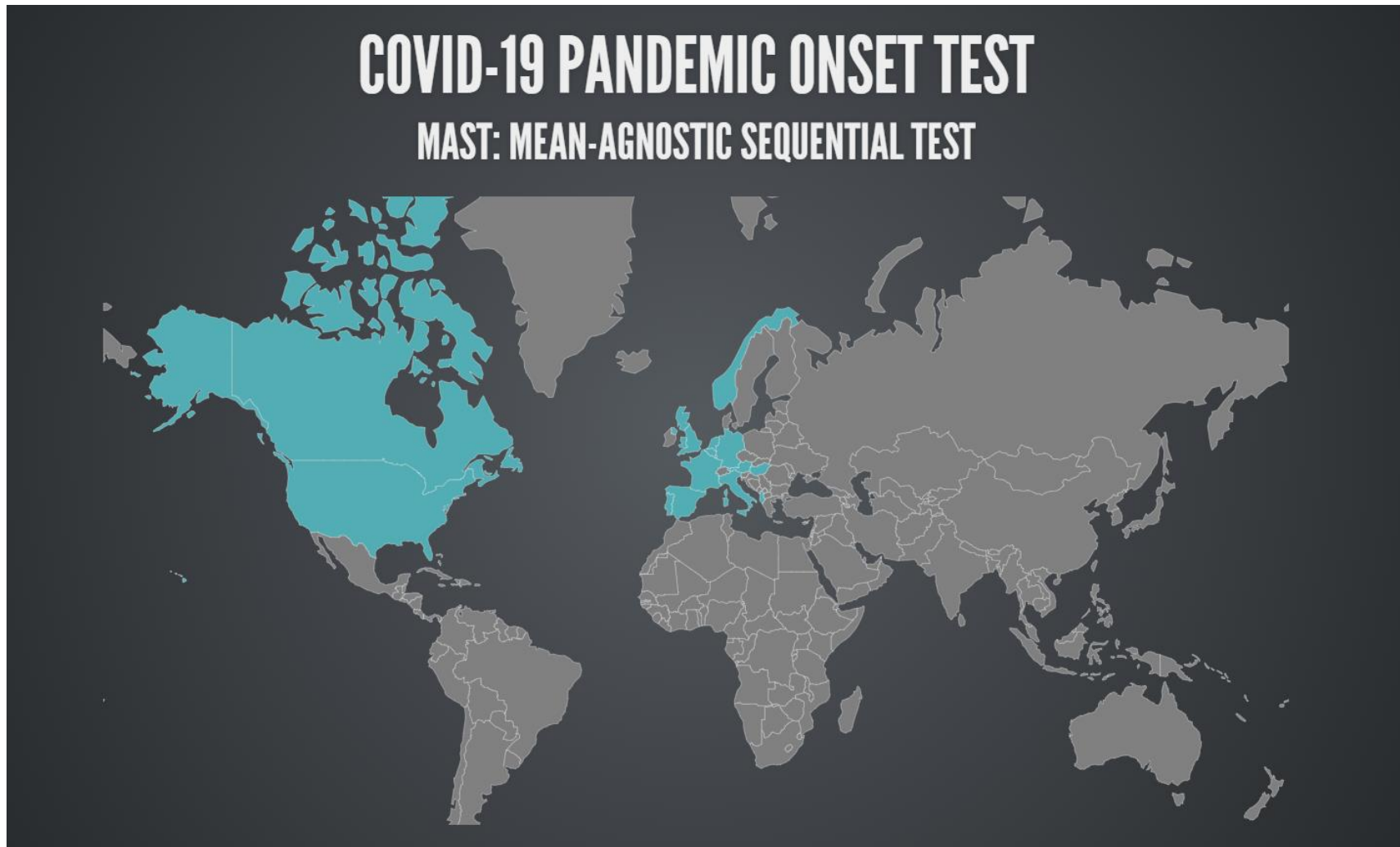

Supplement: Supplementary file 2 — Supplementary Information 2. [file 41598_2021_86827_MOESM2_ESM.pdf]
